# Evaluation of the Immunogenicity of a Vascular Graft Covered with Collagen Derived from the European Carp (*Cyprinus carpio*) and Bovine Collagen

**DOI:** 10.1155/2019/5301405

**Published:** 2019-02-28

**Authors:** Lukas Lambert, Michaela Novakova, Peter Lukac, Dana Cechova, Lenka Sukenikova, Jiri Hrdy, Mikulas Mlcek, Hynek Chlup, Tomas Suchy, Tomas Grus

**Affiliations:** ^1^Department of Radiology, First Faculty of Medicine, Charles University and General University Hospital in Prague, U Nemocnice 2, 128 08 Prague 2, Czech Republic; ^2^Institute of Immunology and Microbiology, First Faculty of Medicine, Charles University and General University Hospital in Prague, Studnickova 7, 128 00 Prague 2, Czech Republic; ^3^Department of Cardiovascular Surgery, First Faculty of Medicine, Charles University and General University Hospital in Prague, U Nemocnice 2, 128 08 Prague 2, Czech Republic; ^4^Institute of Physiology, First Faculty of Medicine, Charles University in Prague, Albertov 5, 128 00, Prague 2, Czech Republic; ^5^Department of Mechanics, Biomechanics and Mechatronics, Faculty of Mechanical Engineering, Czech Technical University in Prague, Technicka 4, 166 07 Prague 6, Czech Republic; ^6^Institute of Rock Structure and Mechanics of the Czech Academy of Sciences, V Holesovickach 41, 182 09, Prague 8, Czech Republic

## Abstract

**Aim:**

To assess the systemic and local immunological response to subcutaneous implants of a vascular graft covered with collagen extracted from the European carp (freshwater fish) or with collagen of bovine origin.

**Methods:**

Pieces of a vascular graft covered by pure bovine (Bos taurus, BOV, n=14) or carp (Cyprinus carpio, CYP, n=14) collagen 5 mm in size were implanted subcutaneously in the dorsum of a Balb/cOla mice. A sham operation group of 12 animals served as the control. At 7 and 14 days after the operation, one-half of each group was terminated and blood for serum, spleen, and implant with surrounding tissue were collected. Mean cytokine (TNF-*α*, IL-10, IL-4, IL-1*β*, IL-13, and IFN-*γ*) levels in serum were determined using ELISA. Spleen cell cultures were used for* in vitro* testing of lymphocyte proliferation and cytokine secretion. Local expressions of IL-6, IL-10, TNF-*α*, TGF-*β*, CCL-2, and CCL-3 were determined using PCR.

**Results:**

We found no significant difference among control, BOV, and CYP groups in mean cytokine serum levels at seven days. At day 14, the BOV group had higher levels of TNF-*α* (P=.018) and both the BOV and CYP groups had lower levels of IL-4 (P=.011 and P=.047, respectively) compared with the control group. Both tested implants showed only a minimal effect on the production of selected cytokines. Cell proliferation in the CYP group stimulated by CYP gel at 14 days was significantly lower than by BOV gel in BOV group (P=.0031) or by CYP gel in the control group (P=.041). The difference between the groups in the local RNA expression of all the tested mediators both at 7 and at 14 days was not significant apart from a lower level of TNF-*α* in the BOV group compared to CYP at 14 days (P=.013).

**Conclusions:**

Implants covered with carp collagen induce an immunological response that is comparable to that of bovine collagen covered implants in a mouse model.

## 1. Introduction

Due to the limited availability of autologous veins for the revascularization of lower limbs in patients with peripheral arterial disease, there is a growing demand for prosthetic bypasses with comparable long-term patency rates even in low-flow conditions [1, 2]. Expanded polytetrafluoroethylene (ePTFE) grafts have become the second best option and widely accepted alternative to the autologous vein with further improvements towards their performance [3].

To overcome the limitations of ePTFE grafts especially in low-flow reconstructions, an effort has been made to engineer and evaluate new materials including collagen. Collagen-impregnated Dacron graft showed similar or even better patency rates in above-knee reconstructions compared to ePTFE grafts in clinical trials [4, 5]. Further development targeting the microstructure of the graft wall offered bi-, tri-, and quadri-laminal designs of the grafts with a scaffold mesh coated by specific fibers, mostly analogs of collagen and elastin, in order to mimic the natural structure of the wall and manipulate its mechanical properties, porosity, and surface properties of the inner layer to minimize the risk intimal hyperplasia or thrombosis [6–8]. However, these innovative grafts underwent limited clinical testing and there has not been a clear example of their bedside success [9].

Collagen is a natural polymer that has immunological properties that play a role in the incorporation of the graft into the body on the outer side and endothelization on the inner side [10]. Common sources of collagen for medical purposes include bovine and porcine tissues, but recombinant human collagen is available as well. Recently, collagen from marine life forms and freshwater fish has been increasingly utilized due to their wide availability and suggested immunomodulatory properties [11]. To our best knowledge, no previous study compared the immunological response to collagen of bovine and freshwater fish origin.

The implantation of biomaterials always initiates an acute inflammatory response that can lead to disintegration and demarcation of the implant or persist as a chronic inflammatory process. The cascade of reactions by the host to the implanted material called the foreign body reaction is essentially an inflammatory response and persists as long as the implanted material is present. This reaction is influenced by the type of the implant, by the site of implantation, and it is species-dependent [12]. Therefore, assessing the nature, duration, and intensity of the immune response against the implanted material and its comparison with other known materials is essential to evaluate its biocompatibility [11].

An inflammatory response is induced at any site that has been damaged. Chemokines and cytokines regulate leukocyte migration and activation, differentiation, and an expression of adhesion molecules on leukocytes and endothelial cells. The production of proinflammatory and regulatory cytokines correlates with the intensity of the inflammatory response. Therefore, it can be used as a marker of its severity and extent [11, 13].

The aim of this study was to assess the systemic and local immunological response to subcutaneous implants of a vascular graft covered with collagen extracted from the European carp (freshwater fish) or with collagen of bovine origin.

## 2. Materials and Methods

This study was approved by the institutional Animal Care and Use Committee and conducted in accordance with National Act No. 246/1992 Coll. as amended on the protection of animals against cruelty that is harmonized with the legislation of EU.

In this experimental study, we compared the systemic and local inflammatory response to subcutaneous implants of a vascular graft covered with pure bovine or carp (*Cyprinus carpio*) collagen in an animal model.

### 2.1. Animals

The animal model chosen for this study was Balb/cOla inbred female mouse (AnLab, Czech Republic). The mice were 8 weeks old at the beginning of the experiment and were housed under specific-pathogen-free conditions with free access to food and water and 12 hours dark/12 hours light cycle.

### 2.2. Implant

**Figure 1:**
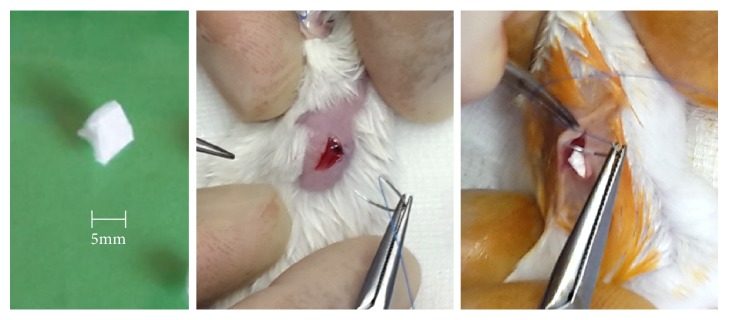
A square piece obtained from the collagen-coated vascular graft (left). Operation in the sham group (middle) and the experimental group (right).

A sterile square piece (size, 5 × 5 mm; weight, 17 mg, Figure 1) of a vascular graft covered by pure bovine (*Bos taurus*) or carp (*Cyprinus carpio*) collagen (DCM Collagen, Czech Republic) was used as the implant in BOV (14 animals) or CYP (14 animals) groups, respectively [2, 14]. A sham operation control group (C) of 12 animals and no implant served as the control.

Briefly, the vascular graft had a three-layer structure and an internal diameter of 6 mm. The scaffold (middle layer) was manufactured from a polyester knitted mesh which was covered with collagen from the inner and outer sides using an extrusion device [2]. Both grafts were extruded from 8 wt% aqueous collagen dispersion.

### 2.3. Implantation

Each animal was shaved at its dorsum one day before the procedure. General anesthesia was introduced with 2% isoflurane and maintained with 1.5% isoflurane inhalation. Then the operation field was swabbed with iodopovidone (Betadine, Mundipharma, Switzerland). A small incision was created and the implant (marked with 5-0 Prolene filament) was inserted subcutaneously 1 cm away from the incision site (Figure 1). The incision was then closed with Prolene 5-0 (Ethicon) suture. In addition to the two experimental groups (BOV, CYP), twelve mice (C) underwent a sham operation to control for the effect of wound healing on the inflammatory reaction.

### 2.4. Postoperative Procedures

Each mouse was moved to a recovery area and monitored. Once recovered, the animals were returned to the cage and observed daily for overall health, macroscopic changes of the wound, and signs of pain.

Seven days after implantation, one-half of the animals from each group were selected for termination. The rest of the animals were sacrificed and analyzed 14 days after implantation. The mice were sacrificed by exsanguination under deep Narcotan anesthesia. The collected samples included blood for serum, spleen, and implant with surrounding tissue.

### 2.5. Serum Cytokine Levels

Mean cytokine levels in serum of experimental groups (BOV, CYP) and control group (C) were determined using an enzyme-linked immunosorbent assay (ELISA) 7 days and 14 days after surgery. To measure the concentration of IL-1*β*, IL-4, IL-10, IL-13, TNF-*α*, and IFN-*γ*, an ELISA kit (Duoset,* RD Systems*, USA) was used according to the manufacturer's instructions.

### 2.6. Tissue Culture

Spleen cell cultures were used for* in vitro* testing of lymphocyte proliferation and cytokine secretion. Mouse mononuclear leukocytes (MML) were isolated from individual spleens by homogenization in a glass tissue grinder and by centrifugation. All cell cultures were conducted in RPMI-1640 medium with L-glutamine and sodium bicarbonate, 40 mg/l gentamicin, 0.002 mol/l HEPES (hydroxyethyl-piperazineethanesulfonic acid), and 5% FCS (fetal calf serum) for both cytokine secretion and proliferation test (*Sigma-Aldrich*, USA). The number of viable cells present in cultures of mouse splenocytes was determined with trypan blue exclusion method. Equal parts of 0.4% trypan blue dye and cell suspension were mixed, incubated for 2 minutes, and immediately counted. Cell viability was good, above 85%.

### 2.7. Cytokine Production Analysis

Cultures of mouse splenocytes (1.1 × 10^6^ per well of 1.9 cm^2^) were incubated in 1 ml of a supplemented RPMI 1640 medium alone (negative control), with pokeweed mitogen (PWM; 1 *μ*g/ml; positive control; Sigma-Aldrich, USA), with bovine and carp gel (1*μ*g/ml), and with bovine and carp implant (0.5mg/ml). The cultures were incubated at 37°C in a humid atmosphere with 5% CO_2_ in 24-well plates for 72 hours; then culture supernatants were harvested and the concentration of IL-1*β*, IL-4, IL-10, IL-13, TNF-*α*, and IFN-*γ* was determined using ELISA kit (Duoset,* RD Systems*, USA) according to the manufacturer's instructions.

### 2.8. Cell Proliferation Test (Blast Transformation Test)

Cell proliferation was performed in 96-well polystyrene flat-bottomed microcultivation plates (*Gama, *Czech Republic) with 2 × 10^5^ cells in 0.25 ml per well. Stimulators were added in the volume of 50 *μ*l. PWM was used as a positive control in a concentration of 1 *μ*g/ml. Splenocyte cultures were stimulated with bovine and carp gel (both in the same concentration of 1 *μ*g/ml), and with 0.5 mg bovine and carp implant pieces per 1 ml of cell culture.

After 48 h incubation, an 18 h pulse of ^3^H-thymidine (37 kBq;* Lacomed*, Czech Republic) was added. The cultures were harvested on a glass fiber Filtermat A (*Wallac Oy*, Belgium) by a 96 Mach 3 Harvester (*Tomtec*, Germany) and counted with 1450 MicroBeta counter (*Wallac Oy*, Belgium) using melt-on scintillator 1450-441 Metilex A (*Wallac Oy*, Belgium).

### 2.9. Local Expression of Cytokine RNA

For detailed characterization of microenvironment from the site of implantation, we analyzed and compared local expression of IL-6, IL-10, TNF-*α*, TGF-*β*, CCL-2, and CCL-3.

### 2.10. RNA Isolation

The small piece of tissue from the place of implantation was removed from each mouse, placed into RNA Stabilization Reagent* (Qiagen*, USA) and frozen at −80°C for later analysis. Later, total RNA was extracted from explanted tissue using RNeasy Fibrous Tissue Mini Kit (*Qiagen*, USA) designed for optimal lysis of fiber-rich tissues and purification of high-quality total RNA. Briefly, 25-30 mg tissue was homogenized with Ultra-Turrax T8 homogenizer (*IKA*, Germany) and incubated with proteinase K at 55°C for 10 minutes. Then RNA was isolated following the manufacturer's instructions. The purity of the RNA was assessed by the ratio of absorbance at 260 and 280 nm. RNA purity was within the range of 2.0–2.1. The total RNA concentration was estimated by spectrophotometric measurements at 260 nm assuming that 44 *μ*g of RNA per milliliter equals one absorbance unit. RNA was stored in aliquots at −20°C until used for reverse transcription.

### 2.11. Real-Time PCR

RNA was converted to cDNA using Taq-Man reverse transcription reagents (*Applied Biosystems*, USA). One microgram of total RNA was used to generate cDNA and then 50 ng of a cDNA was used for quantitative PCR analysis for each reaction. The PCR reaction of 40 cycles was performed using primers of interleukins (IL-6, IL-10), tumor necrosis factor alpha (TNF-*α*), transforming growth factor beta (TGF-*β*), monocyte chemoattractant protein 1 (MCP-1/CCL-2), macrophage inflammatory protein 1*α* (MIP-1*α*/CCL-3), and the control gene *β*-actin (*Applied Biosystems*, USA).

The final reaction volume of 25 *μ*l contained 20 *μ*l of reaction mix and 5 *μ*l of cDNA, each sample was analyzed in duplicate. The PCR reaction was run on a 7300 real-time PCR System (*Applied Biosystems*, USA) under standard conditions.

### 2.12. Statistical Analysis

Statistical tests were performed using Prism 5.0 (GraphPad Software, San Diego, CA, USA) and R (R Core Team, Vienna, Austria). We used ANOVA with Tukey's HSD post hoc tests and the* t*-test to test for statistical significance. A p-value below 0.05 was considered significant.

## 3. Results

### 3.1. In Vivo Observation

All animals survived the surgical procedure and thrived well and did not show any signs of depression or pain and there were no complications of wound healing.

### 3.2. Serum Cytokine Levels

**Figure 2:**
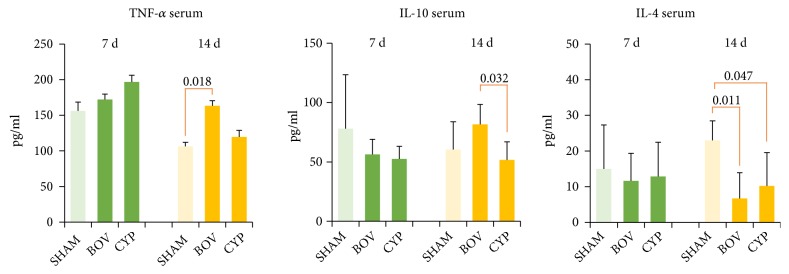
Mean cytokine levels (pg/ml) in serum of experimental groups (BOV, CYP) and control group (SHAM) determined using an enzyme-linked immunosorbent assay (ELISA) 7 days (green bars) and 14 days (yellow bars) after surgery.

The concentration of the serum cytokines is presented in Figure 2. We found no difference among control, BOV, and CYP groups in mean serum cytokine levels (TNF-*α*, IL-4, and IL-10) at seven days after the surgery. At day 14, the BOV group had higher levels of TNF-*α* (163±34pg/ml vs. 107±27pg/ml, P=.018), the level of IL-10 was higher in the BOV group compared to the CYP group (81.6±18.1pg/ml vs. 51.7±16.5pg/ml, P=.032), and both the BOV and CYP groups had lower levels of IL-4 (6.7±7.2pg/ml and 10.2±9.3pg/ml vs. 23.0±5.4pg/ml, P=.011 and P=.047, respectively) compared to the control group. Cytokine levels of IL-1*β*, IL-13, and IFN-*γ* were below the detection limit.

### 3.3. Cytokine Production Analysis

**Figure 3:**
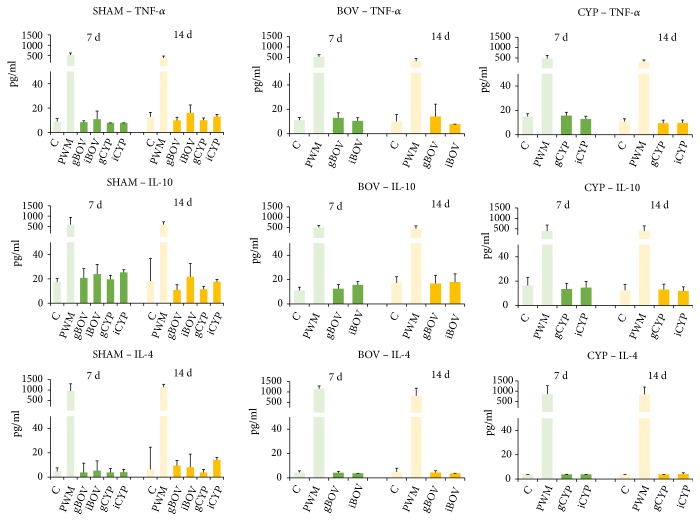
Mean cytokine levels (pg/ml) in supernatants of* in vitro* stimulated splenocytes of implanted (BOV, CYP) and control groups (SHAM) determined using an enzyme-linked immunosorbent assay (ELISA) 7 days (green bars) and 14 days (yellow bars) after surgery. Cultures of mouse splenocytes were incubated in supplemented RPMI 1640 medium alone (C, negative control), with pokeweed mitogen (PWM, positive control), with bovine and carp gel (gBOV or gCYP), and bovine and carp implant (iBOV or iCYP). After a 72-hour incubation, the cultures were centrifuged and cytokine expression was measured in supernatants. All differences are not significant.

Both tested implants showed only a minimal effect on the production of selected cytokines. Cytokine levels of IL-1*β*, IL-13, and IFN-*γ* were below the detection limit (≤ 7 pg/ml) in both the serum and culture supernatant. Furthermore, there was no significant difference among the cytokine responses of sham-operated control mice and response to collagen-implanted mice of both groups in cytokine levels of TNF-*α*, IL-10, or IL-4 (Figure 3).

### 3.4. Cell Proliferation (Blast Transformation Test)

**Figure 4:**
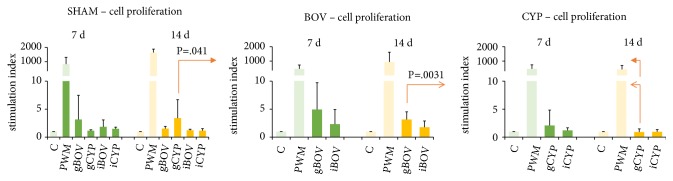
Cell proliferation of* in vitro* stimulated splenocytes of implanted (BOV, CYP) and control groups (SHAM) determined 7 days (green bars) and 14 days (yellow bars) after surgery expressed as stimulation index. Cultures of mouse splenocytes were incubated in supplemented RPMI 1640 medium alone (C, negative control), with pokeweed mitogen (PWM, positive control), with bovine and carp gel (gBOV or gCYP), and bovine and carp implant (iBOV or iCYP). After 48 h incubation, an 18 h pulse of ^3^H-thymidine (37 kBq) was added. Its incorporation by mouse lymphocytes was measured by a beta-counter. The results are expressed as stimulation index - mean activity (count per minute) relative to the negative control (K). Cell proliferation in the CYP group by CYP gel at 14 days was significantly lower than by BOV gel in BOV group (P=.0031) or by CYP gel in control group (P=.041).

Cell proliferation in the CYP group stimulated by CYP gel at 14 days was significantly lower than by BOV gel in BOV group (P=.0031) or by CYP gel in control group (P=.041, Figure 4).

### 3.5. Local Expression of Cytokine RNA

**Figure 5:**
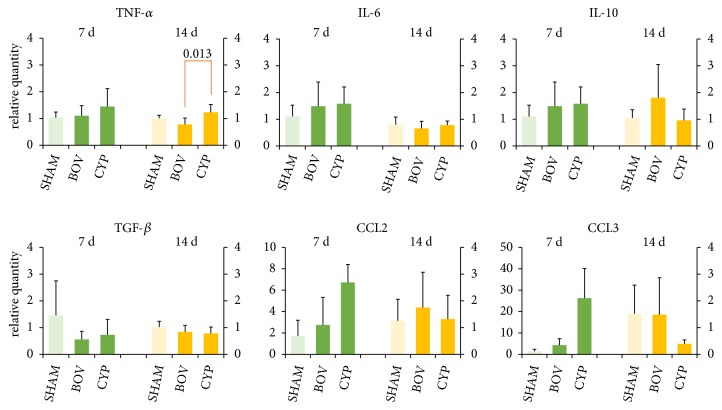
RNA relative quantity (RQ) in implant surrounding micro-environment tissue. Results are calculated from normalized gene expression data. The RQ values indicate the difference between the implanted group (BOV, CYP) and controls (SHAM). For each group, the RQ values are charted as average ± SD. Statistical analysis showed decreased TNF-alpha in BOV at 14 days.

We found no significant difference between the control group and the BOV or CYP groups in the expression of IL-6, IL-10, TGF-*β*, or CCL-2 both at 7 and at 14 days after implantation (Figure 5). At 14 days, TNF-*α* levels were higher in the CYP group (1.23±0.30) compared to the BOV group (0.76±0.24, P=.013).

## 4. Discussion

This experimental study showed that implant of a vascular graft covered with carp collagen has at least comparable immunogenicity with regard to circulating cytokine levels, stimulated cytokine production and cell proliferation, and local expression of cytokine RNA as graft covered with bovine collagen in a mouse model.

In medicine, collagen is mostly used as wound dressing material, as a scaffold for tissue regeneration, or as a biodegradable mechanical support for hollow viscus (stents) or in orthopedics (screws) [15, 16]. The process of extraction of collagen fibers has repeatedly been described in numerous species including marine life forms and freshwater fish [17, 18]. Apart from pure mechanical support, fish collagen known for its low antigenicity also promotes spatial cell organization by intercellular signaling and cell adhesion and modulates local inflammatory reaction [19, 20]. Intravascular deployment of collagen-based or coated structures is more challenging. Beside programming mechanical properties by the arrangement of the fibers and supporting scaffold, antigenicity and thrombogenicity have to be rigorously tested [21].

To determine the nature and intensity of the inflammatory response to the implant covered with carp collagen and compare it to that induced by bovine collagen covered implant, we chose albino mice BALB/c strain, which has been previously shown to be responsive to collagens [22, 23]. We measured the serum level of proinflammatory and anti-inflammatory cytokines (IL-1*β*, IL-4, IL-10, IL-13, TNF-*α*, and IFN-*γ*), their secretion by* in vitro* stimulated splenocytes, proliferating activity of* in vitro* stimulated splenocytes, and local production of selected cytokine (TNF-*α*, IL-6, IL-10, TGF-*β*) and chemokine (CCL2 and CCL3) mRNA as previously done by our group and others [11, 13].

In general, many chemokines (e.g., CCL2 and CCL3) are involved in the recruitment of inflammatory macrophages, and cytokines (e.g., IL-1*β*, IL-4, IL-6, IL-10, IL-13, IFN-*γ*, and TGF-*β*) determine the behavior of macrophages through different pathways of activation. Furthermore, the cytokines IL-4 and IL-13 can induce multiple fusogenic effects, which result in membrane fusion and giant cell formation and are responsible for the polarization of immunity towards Th2 response and IgE mediated allergy [24]. In addition, many of these mediators are also involved in the regulation of proteolysis and cellular phagocytosis of collagen [12].

Values of serum TNF-*α* in naïve mice depend on the mouse strain, analytic technique, and even housing conditions. According to some authors, serum level of TNF-*α* in healthy unstimulated mice is low (≤ 20 pg/ml, ELISA test) [25, 26]. But we and also others [27, 28] measured higher TNF-*α* values (range 100-200pg/ml) in the sera of the control mice. Furthermore, in our study, serum levels of TNF-*α* were similar in each group of mice no matter what group (control or implanted) it was. It must be noted that control mice underwent all procedures as implanted animals, including surgery, and that all animals were doing well after the surgery. There was no difference among the groups. Higher levels of TNF-*α* are found in inflammatory and infectious conditions; it has been confirmed that serum and tissue levels of this cytokine correlate with the severity of the inflammation. Values of serum TNF-*α* in stimulated mice can reach up to 1-10 ng/ml [29].

Based on our pilot experiments, we decided to assess the immune response at day 7 and day 14 after the implantation, as we observed high activity of systemic response in these days that was later decreasing [30]. We focused on monitoring the immune response to implant at the following time points after implantation that were related to the distinct phases of the foreign body reaction in mice: one week (early progression) and two weeks (intermediate progression) [12]. We did not monitor the early phase of the response as the focus of our interest was a specific response to collagen, which develops up to several days after activation. Furthermore, this onset phase of response is strongly influenced by surgery.

The spectrum of tested cytokines provides basic orientation in the reactivity of the organism to the implant [22, 30]. Very low levels of both pro- and anti-inflammatory cytokines in serum and in supernatants of stimulated splenocytes are indicative of a good tolerance of the implant. Lower concentrations of IL-4 in serum in BOV and CYP group and at 14 days compared to the sham group and lower proliferation activity of splenocytes stimulated by the carp collagen compared both to the sham and BOV groups at 14 days may be explained by the immunomodulatory effect of the collagen which is more pronounced in the CYP type described by other authors [19, 20].

The difference in the production of TNF-*α* RNA in the microenvironment surrounding the BOV and CYP implants at two weeks should be regarded in the perspective of the low levels of other mediators in all groups (including sham) and the absence of any significant difference between the sham and CYP groups as a merely faster decrease of this cytokine in the BOV group.

In general, our findings of low immunological reactivity to fish collagen in an animal model are in agreement with previously conducted research on the biocompatibility of collagen extracted from marine and freshwater sources by other research groups [11, 31]. Immunological response to collagen material depends on collagen source as well as the processing of the collagen product including crosslinking, softening, and sterilization. For these reasons, we tested the reactivity of mice to the final prosthetic graft [32].

Because implants impregnated with the carp collagen induce only a mild immune reaction in mice or even slightly downmodulate the reaction, we believe that they can be a substitute to bovine collagen in the future. Further preclinical evaluation of the implants is warranted to assess whether carp collagen coated vascular prostheses influence thrombogenicity and patency in the long term. On a macroscopic level, the mechanical stability of the implants and their integration into the body need to be studied as well [2, 30].

This study has several limitations. Firstly, we attempted to minimize the number of study animals, which were divided into a total of six groups (sham, BOV, CYP) terminated at 7 and 14 days. Secondly, we used only a selected range of methods for the analysis of the immunological response. Other methods such as western blotting or immunofluorescence microscopy could have contributed to a better insight into the data. Thirdly, the research was performed in an animal model and the results cannot be generalized to humans. Lastly, the assessment of biocompatibility was performed only in the short term.

## 5. Conclusions

Vascular implants covered with carp collagen induce a minimal immunological response that is comparable to that of bovine collagen covered implants. These findings pave the way for the design and preclinical evaluation of a carp collagen coated vascular implants.

## Data Availability

All data are included in the manuscript.
